# Tumor Stress-Induced Phosphoprotein1 (STIP1) as a Prognostic Biomarker in Ovarian Cancer

**DOI:** 10.1371/journal.pone.0057084

**Published:** 2013-02-27

**Authors:** Angel Chao, Chyong-Huey Lai, Chia-Lung Tsai, Swei Hsueh, Chuen Hsueh, Chiao-Yun Lin, Hung-Hsueh Chou, Yu-Jr Lin, Hsi-Wen Chen, Ting-Chang Chang, Tzu-Hao Wang

**Affiliations:** 1 Department of Obstetrics and Gynecology, Chang Gung Memorial Hospital and Chang Gung University, Taoyuan, Taiwan; 2 Department of Clinical Pathology, Chang Gung Memorial Hospital and Chang Gung University, Taoyuan, Taiwan; 3 Biostatistical Center for Clinical Research, Chang Gung Memorial Hospital, Taoyuan, Taiwan; 4 Graduate Institute of Biomedical Sciences, Chang Gung University, Taoyuan, Taiwan; 5 Genomic Medicine Research Core Laboratory, Chang Gung Memorial Hospital, Taoyuan, Taiwan; Duke University Medical Center, United States of America

## Abstract

Stress-induced phosphoprotein 1 (STIP1) has been recently identified as a released biomarker in human ovarian cancer. In addition, STIP1 secreted by human ovarian cancer cells has been shown to promote tumor cell proliferation by binding to ALK2 (activin A receptor, type II-like kinase 2) and activating the SMAD-ID3 signaling pathways. In this study, a total of 330 ovarian cancer tumor samples were evaluated for STIP1 expression by immunohistochemistry and analyzed for a possible correlation with patient characteristics and survival. The quantification of immunoreactivity was accomplished by applying an immunohistochemical scoring system (histoscore). Patients with high-level STIP1 expression (histoscore ≥169) had a significantly worse survival (high STIP1, mean survival time = 76 months; low STIP1, mean survival time = 112 months; *P*<0.0001). Moreover, STIP1 histoscores were significantly higher in high-grade tumors (grade 3) than in low-grade (grade 1–2) malignancies (*P*<0.0001), suggesting that STIP1 may be a proxy for tumor aggressiveness. The results of multivariable analysis revealed that high STIP1 histoscores, advanced stages, histologic types, and the presence of residual disease (≥2 cm) were independent predictors of poor prognosis. The addition of STIP1 histoscores improved the prediction of overall and progression-free survival rates in the multivariable Cox proportional hazard model. The treatment of ovarian cancer cells with recombinant STIP1 stimulated cell proliferation and migration, but co-treatment with anti-STIP1 antibodies abrogated this effect. Our findings suggest that STIP1 expression may be related to prognosis and that the STIP1 pathway may represent a novel therapeutic target for human ovarian cancer.

## Introduction

Epithelial ovarian cancer is one of the most lethal malignancies affecting women [1]. Nearly 225,500 ovarian cancer new cases are occurring worldwide each year, responsible for 140,200 deaths [2]. Ovarian carcinomas comprise a heterogeneous group of neoplasms, the four most common histological subtypes being serous, endometrioid, clear cell, and mucinous [3]. The measurement of serum cancer antigen 125 (CA125) levels has become standard practice for the preoperative evaluation of ovarian masses [4]. In addition, CA125 has been shown to be useful for monitoring therapeutic response and in the surveillance of patients with epithelial ovarian cancer [5]. However, CA125 is not elevated in all ovarian tumors and does not have sufficient positive predictive value for population-based risk assessment or early detection [6]. Due to the limitations of CA125 as a disease marker, there is an urgent need for new biomarkers that can be used as prognostic indicators in ovarian cancer to effectively differentiate between aggressive and less aggressive disease.

Recent technological advances, especially in the fields of genomics and proteomics, are expediting the discovery of new cancer biomarkers [7], [8]. By comparing the proteomes of the tumor interstitial fluid (TIF) and the normal interstitial fluid (NIF), we have recently identified stress-induced phosphoprotein 1 (STIP1) as a candidate biomarker for human ovarian cancer [9]. Serum STIP1 levels are significantly higher in patients with ovarian cancer than in age-matched healthy controls [9] and decrease significantly after surgical removal of the tumor [10]. In addition, the combined measurement of CA125 and STIP1 has been shown to improve the early detection of ovarian cancer [9], supporting the clinical usefulness of STIP1 as a biomarker in this malignancy [11].

STIP1, also known as Hsp70/Hsp90-organizing protein (HOP), transformation-sensitive protein IEF SSP 3521 (IEF-SSP-3521), P60, STI1, STI1L, (GeneID 10963; HPRD 05454), a 62.6 kDa protein, contains three tetratricopeptide repeat (TPR) domains [12] which are able to interact with heat shock proteins to form complexes that participate in diverse biological processes ranging from RNA splicing, transcription, protein folding, signal transduction, and cell cycle regulation [13], [14]. In neuronal tissues, extracellular STIP1 binds to prion proteins and triggers different signaling pathways – including the endogenous mitogen-activated protein kinase 1/2 (ERK1/ERK2), protein kinase A, and phosphatidylinositol 3-kinase signaling cascades – ultimately leading to an increase in cell proliferation [15], [16], [17]. In ovarian cancer, we have shown that STIP1 binds to a bone morphogenetic protein (BMP) receptor – termed activin A receptor, type II-like kinase 2 (ALK2) – to activate the SMAD signaling pathway and the transcriptional activation of ID3 (inhibitor of DNA binding 3) [10]. Taken together, these findings suggest that STIP1 secreted by human ovarian cancer cells promotes cancer cell proliferation by acting in an autocrine and/or paracrine fashion.

Although the release of STIP1 from human ovarian cancer has been confirmed by an independent research group [18], few data are available on the usefulness of STIP1 immunohistochemical analysis for assessing prognosis in ovarian cancer [19]. In this study, a total of 330 ovarian cancer tumor samples were evaluated for STIP1 expression by immunohistochemistry and analyzed for a possible correlation with patient characteristics and survival.

## Materials and Methods

### Ethics Statement

This study was conducted in accordance with the Helsinki declaration and was approved by the Institutional Review Board of the Chang Gung Memorial Hospital (CGMH-IRB #99-0112B and #97-1444C).

### Patients

Between 2000 and 2005, a total of 403 consecutive patients diagnosed with ovarian cancer at the Linkou Medical Centre of Chang Gung Memorial Hospital were included in the study. The histological types were as follows: serous (n = 160), mucinous (n = 68), endometrioid (n = 73), clear cell carcinoma (n = 64), and mixed cell type (n = 38). The exclusion criteria were as follows: (i) patients who underwent neoadjuvant therapy before definite surgery or who were referred from outside hospitals after the initial surgery; (ii) patients who underwent laparoscopic surgery as the primary treatment; (iii) patients who were not followed-up in the first three months after primary treatment; (iv) patients with undifferentiated carcinomas arising in teratomas; and (v) patients with unavailable pathological paraffin blocks. Progression-free survival (PFS) was calculated as the time interval (in months) from the date of surgery to the date of documented disease progression (retrospectively defined based on the elevation of serum CA125 and/or radiographic evidence of progression). Overall survival (OS) was defined as the time interval between the date of surgery and the date of death.

### Immunohistochemistry

The archives of formalin-fixed, paraffin-embedded ovarian cancer tissue sections were retrieved as described previously [9], [20], [21]. The sections were stained with a primary mouse anti-human STIP1 monoclonal antibody (Abnova Corp., Taipei, Taiwan; 1∶1,800 dilution) using an automated immunohistochemistry stainer with the Ventana Basic DAB (3,3-diaminobenzidine) Detection kit (Tucson, AZ, USA). The slides were evaluated independently by two pathologists (S.H. and C.H.) who were blinded to the clinicopathological data and the patients’identities. The overall immunohistochemical score (histoscore) was expressed as the percentage of positive tumor cells (0−100%) multiplied by their staining intensity (0 = negative, 1 = weak, 2 = moderate, 3 = strong). Therefore, the total histoscore ranged from 0 to 300 [22]. The validation of the anti-STIP1 antibody specificity was performed by blocking the antibodies with 400 nM of recombinant human STIP1 protein (rhSTIP1) during the incubation step with anti-STIP1.

### Cell Culture

Ovarian cancer cell lines (BG1, MDAH2774) were obtained from American Type Culture Collection (Manassas, VA, USA). Cells were cultured in 10% FBS, penicillin (100 units/mL), streptomycin (100 units/mL), and DMEM/F12 media at 37°C in a 5% CO_2_ atmosphere.

### Cell Migration Assay

BG1 and MDAH2774 cells (10^6^/well) − treated with either rhSTIP1 (400 nM) or the vehicle alone − were cultured in serum-free medium for 24 h. Cells were then plated into the upper chamber of a Transwell (24-well, 8-µm pore size; Corning Inc., Corning, NY, USA). The lower chamber was filled with 800 µL of DMEM/F12 and 0.5 µg/mL of fibronectin (Sigma, St. Louis, MO, USA). After 26 hours of incubation, the cells that had migrated through pores and adhered to the lower membrane were stained with fluorescein and calcein-AM (4 µg/mL; BD Biosciences, San Diego, CA, USA). The number of viable cells that had traversed the filter was determined by fluorescence measurement of each sample using a Tecan Infinite M200 Multiwell reader (Tecan, Männedorf, Switzerland). The neutralization of STIP1 was performed using an anti-STIP1 antibody (800 nM). All assays were repeated at least three times.

### Wound Closure Assay

To investigate the effect of STIP1 on cell migration, MDAH2774 cells (10^6^/well) that were either treated with 400 nM of rhSTIP1 or the vehicle were seeded in 3.5-cm dishes after 24 h of culture in serum-free medium. The medium was then replaced with one that contained 0.4% FBS. Cell migration was observed through an opening. Phase contrast images of the gaps were captured at baseline and after 24 h of treatment with an inverted microscope (magnification, 10×). The WimScratch software (Wimasis, Munich, Germany) was used for the analysis of wound closure [23].

### Cell Viability Assay using 3-(4,5-Dimethylthiazol-2-yl)-2,5 Diphenyltetrazolium (MTT)

The conversion of the yellow tetrazolium salts to purple formazon crystals by mitochondria was used as a proxy for cell viability. In 48-well plates, 5×10^4^ cells were cultured in 200 µl of medium per well overnight. Twenty µl of MTT reagent was added to each well, and after 4 h, 200 µl of solubilization solution (10% SDS in 0.01 M HCl) per well was added for overnight. Absorbance was determined at 570 nm in a microplate spectrophotometer (PerkinElmer Life Science). For testing the effect of knockdown of STIP1 on cell viability, ovarian cancer cells were transfected with 50 nM of double-stranded siRNA targeting STIP1 or control siRNA in Lipofectamine RNAimas (Invitrogen, Calsbad, CA), as we previously reported [10]. After 48 h of transfection, cells were subcultured in the plate for MTT assays.

### Bromodeoxyuridine (BrdU) Incorporation Assay

To investigate the effect of STIP1 on cell proliferation, BG1 and MDAH2774 cells (10^4^ cells/well) were cultured in complete medium for 24 h, followed by 72 h of serum starvation. The cells were then treated with either 400 nM of rhSTIP1 or the vehicle for 24 h. DNA synthesis was assayed with the Cell Proliferation ELISA, BrdU Kit (Roche Applied Science, Indianapolis, IN, USA) using colorimetric detection according to the manufacturer’s protocol [21], [23]. The neutralization of exogenous STIP1 was performed using the anti-STIP1 antibody (800 nM).

### Ki67 Immunocytochemical Assay

BG1 and MDAH2774 cells were serum starved for 72 h and then treated with either 400 nM of rhSTIP1 or the vehicle for 24 h. The proliferative activity was tested by immunocytochemical staining of Ki67 using a monoclonal antibody against Ki67 (NeoMarker, Fremont, CA, USA).

### Statistical Analysis

The differences in the histoscore between two groups were compared using the Mann-Whitney *U* test. The Kruskal-Wallis test was used for comparing more than two groups. The ability of STIP1 histoscores to discriminate between the survived and the deceased patients was assessed by plotting the receiver operating characteristics (ROC) curve, which associates the true positive rate (sensitivity) to the false positive rate (1-specificity) and by computing the area under the curve (AUC). Time-dependent ROC curves were used to assess the AUCs at different time points [24]. The Cox proportional hazards regression analysis was used to identify the independent predictors of OS and PFS. All of the variables with univariate associations at *P*<0.05 were entered into the multivariable model. To investigate the impact of adding STIP1 to the prognostic Cox model for patients with invasive ovarian cancer, we subtracted the deviance of the model with the addition of STIP1 from the deviance of the model without STIP1. The difference was then tested against a chi-square distribution with degrees of freedom equal to the difference between the degrees of freedom of the two models (either with or without STIP1) [25]. The results are presented as adjusted hazard ratios (HR) and 95% confidence intervals (CI). All analyses were carried out using the SPSS 17.0 statistical package (SPSS Inc., Chicago, IL, USA). We used the R package *survivalROC* (R version 2.15.1; The R foundation for Statistical Computing, Vienna, Austria) for the calculation of time-dependent ROC curves. Two-tailed *P* values <0.05 were considered statistically significant.

## Results

### A High STIP1 Immunohistochemical Expression is Associated with High-stage, High-grade, and Invasive Ovarian Cancer

**Figure 1:**
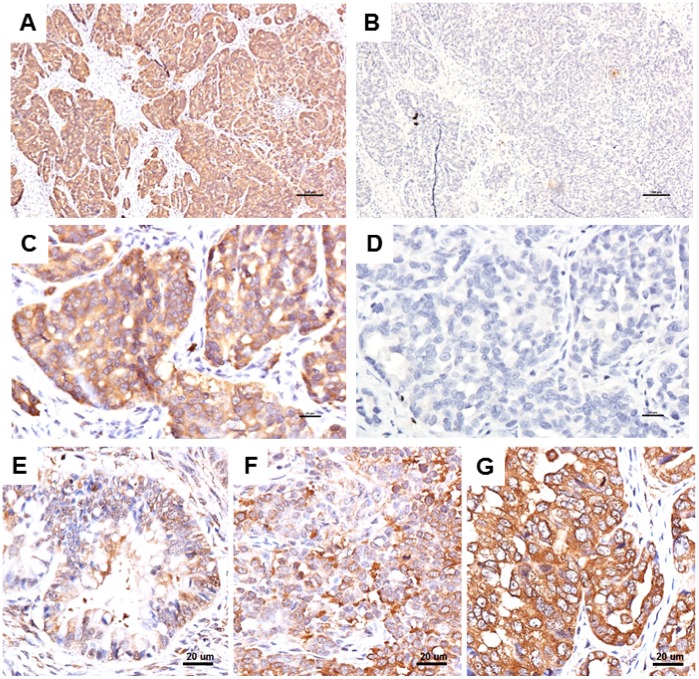
Specificity of the anti-STIP1 antibody (panels A–D) and the intensity of immunostaining for STIP1 (panels E–G). STIP1 expressed in the cytoplasm of ovarian cancer cells is stained brown. The immunoreactivity of STIP1 to the anti-STIP1 antibody in cancer tissues (A, C) was abrogated by the addition of 15 µg of recombinant human STIP1 during incubation (B, D); these results support the specificity of the anti-STIP1 antibody. The intensity of the STIP1 staining was graded as 1 (E), 2 (F), and 3 (G). All of the slides presented here were from ovarian serous carcinomas. The scale bars represent 100 µm (A, B) or 20 µm (C–G).

The results of immunohistochemistry (IHC) showed that the treatment with an excess amount of recombinant STIP1 completely abrogated the immune-recognition of tissue STIP1 by anti-STIP1, confirming the specificity of the anti-STIP1 antibody used in this study (**Figures 1A–D** **)**. For the identical anti-STIP1 antibody used in IHC throughout this study, western blot analysis of multiple ovarian cancer cell lines also confirmed its specificity in recognizing STIP1 (**Figure S1**). The differences in STIP1 immunohistochemical staining are shown in **Figures 1E–G**.

A total of 330 patients with ovarian cancer (median age, 50.7 years; range, 17−90 years) were included in the study. The general characteristics of the study patients are presented in **Table 1**. Of the 50 patients with borderline ovarian tumors (BOT), 49 (98%) were in stage I or II. By contrast, 146 (52.1%) of the 280 patients with invasive cancer were in stage III or IV. Serous carcinoma was the most common histological subtype. Higher STIP1 histoscores were significantly associated with older age (≥50 years), advanced stage, the presence of non-mucinous cancers (serous, clear cell, or endometrioid carcinomas), grade 3, invasive cancer, higher CA125 levels (≥35 U/mL), and suboptimal primary surgical cytoreduction (residual disease ≥2 cm) (**Table 1**).

**Table 1:** Clinicopathological characteristics and STIP1 histoscore^a^ in 330 patients with ovarian cancer.

Characteristic	Patients (n = 330)	STIP1 histoscore	*P* value
**Age, years**			*<0.005
** <50**	156 (47.3%)	160.0±96.8	
** ≥50**	174 (52.7%)	193.5±87.3	
**Stage**			*<0.0001
** I–II**	183 (55.5%)	147.4±95.3	
** III–IV**	147 (44.5%)	215.4±75.4	
**Histological type**			**<0.0001
** Serous**	121 (36.7%)	206.8±79.4	
** Mucinous**	68 (20.6%)	81.3±78.9	
** Clear cell**	57 (17.3%)	190.2±90.6	
** Endometrioid**	66 (20.0%)	201.3±67.1	
** Others**	18 (5.4%)	219.6±8.8	
b **Grade**			*<0.0001
** 1–2**	110 (33.3%)	165.0±83.5	
** 3**	113 (34.2%)	231.3±65.0	
**BOT vs Invasive cancer**			*<0.0001
** BOT**	50 (15.2%)	70.1±69.9	
** Invasive cancer**	280 (84.8%)	196.9±83.4	
**Serum CA125 (U/mL)**			*<0.0001
** <35**	65 (19.7%)	140.4±97.2	
** ≥35**	241 (73.0%)	189.2±87.2	
**Residual disease**			*<0.0001
** <2 cm**	219	158.2±94.5	
** ≥2 cm**	111	216.0±78.0	

* Mann-Whitney U test.

** Kruskal-Wallis test.

a histoscore = percentage × intensity.

b Clear cell carcinoma and borderline malignancies are not graded [3].

BOT, borderline ovarian tumor.

### A High STIP1 Immunohistochemical Expression is Associated with Reduced Overall Survival (OS)

**Figure 2:**
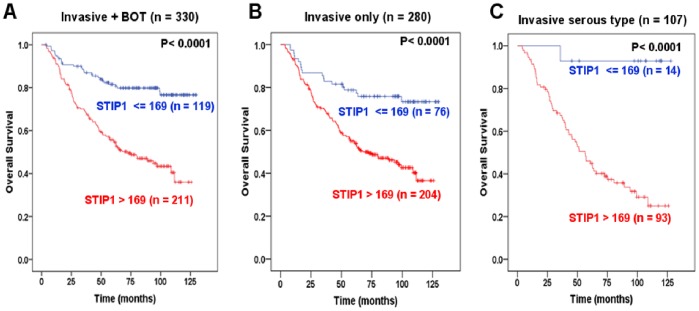
High STIP1 histoscores are associated with a reduced overall survival. Kaplan-Meier curve analyses for overall survival showed that women with a STIP1 histoscore >169 (red line) had a significantly lower overall survival than those with a STIP1 histoscore ≤169 (blue line). Statistical significances (*P*<0.0001) were detected in the analyses of (A) all cases (n = 330) including invasive cancer and borderline tumor (BOT), (B) invasive ovarian cancers of all cell types (n = 280), and (C) only invasive serous type of ovarian cancers (n = 107).

The median duration of the follow-up was 69.3 months (range, 4−130 months). To investigate the potential usefulness of STIP1 immunohistochemical expression as a biomarker in ovarian cancer, we used the ROC curve to quantify how well different histoscores could be used for the prediction of survival. Because a minimum follow-up of 3 years was required for survival analysis determined by the ROC curve, a total of 245 patients were included in this analysis. The optimal cutoff for the STIP1 histoscore to discriminate between patients who survived (n = 191) and those who died (n = 54) was 169 (sensitivity = 0.889, specificity = 0.492). In the analysis of all studied patients including both invasive (n = 280) and borderline cases (n = 50), the cumulative OS of the patients with STIP1≤169 (n = 119) was significantly higher (*P*<0.0001) than those with a score >169 (n = 211) in the Kaplan-Meier analysis. The mean survival periods were 112 and 76 months, respectively **(** **Figure 2A** **)**.

Because borderline ovarian tumors are generally characterized by a good prognosis and are not commonly included in ovarian cancer clinical trials, the independent prognostic significance of STIP1 levels was analyzed only in the subgroup of patients with invasive ovarian cancer (n = 280). The results of multivariate analysis indicated that STIP1 histoscores >169 were independent, significantly prognostic factor for OS (*P*<0.005) and for PFS (*P*<0.05) **(** **Table 2** **)**. Kaplan-Meier analyses also revealed that STIP1 histoscores >169 were significantly associated with poor OS both in all invasive cases of this study (n = 280) (**Figure 2B**) and in invasive serous ovarian cancers (n = 170) (**Figure 2C**).

**Table 2:** Multivariate analyses of clinicopathological parameters for overall and progression-free survivals.

Characteristics	overall survival			progression-free survival		
	HR	95% CI	*P* value	HR	95% CI	*P* value
**Age, years**			0.815			0.483
** <50**	1			1		
** ≥50**	1.05	0.71–1.55		0.88	0.61–1.26	
**Stage**			<0.0001			<0.0001
** I–II**	1			1		
** III–IV**	4.66	2.34–9.25		3.81	2.16–6.72	
**Histological type**			<0.005			0.065
** Serous**	1			1		
** Mucinous**	3.10	1.17–8.22		1.36	0.55–3.40	
** Clear cell**	3.0	1.79–5.03		1.96	1.22–3.16	
** Endometrioid**	1.10	0.61–1.98		1.07	0.65–1.77	
** Others**	1.45	0.77–2.73		1.50	0.82–2.73	
**STIP1 histoscore**			<0.005			<0.05
** <169**	1			1		
** ≥169**	2.45	1.37–4.36		1.81	1.12–2.92	
**CA125 (U/mL)**			0.458			0.351
** <35**	1			1		
** ≥35**	1.47	0.52–4.14		1.50	0.64–3.53	
**Residual disease**			<0.0001			<0.0001
** <2 cm**	1			1		
** ≥2 cm**	2.97	1.78–4.93		3.03	1.92–4.77	

CI: confidence interval; HR, hazard ratio.

### A High STIP1 Immunohistochemical Expression is Associated with High-grade Tumors

Histological grading is an important parameter for the risk assessment of patients with ovarian cancer. As shown in **Table 1**, the STIP1 histoscores were significantly higher in high-grade (grade 3) tumors than in low-grade (grade 1–2) malignancies (*P*<0.0001). We further investigated the clinical significance of STIP1 immunoexpression in ovarian cancers according to their histological type and grade. The majority of grade 3 tumors showed a STIP1 histoscore >169; in particular, 92.8% (77/83) of the serous type, 92.3% (12/13) of endometrioid, and 76.5% (13/17) of the mixed type carcinomas showed a high STIP1 expression. Of the 57 clear cell ovarian cancers which are usually not graded [3], 40 (70.2%) exhibited a STIP1 histoscore >169. The cumulative OS rates of patients with low-grade (grade 1–2) malignancies were significantly higher than those of patients with grade 3 malignancies (*P*<0.0001). The mean survival periods were 102 and 66 months, respectively (**Figure S2**). Taken together, these results indicate that high STIP1 histoscores are associated with a particularly aggressive behavior in ovarian cancer.

### The Addition of STIP1 Histoscores Improves the Prognostic Stratification of Patients with Invasive Ovarian Cancers

To test whether STIP1 histoscores provided information in addition to currently used tumor grading and staging, we analyzed the impact of STIP1 levels on clinical survival only in patients with the same grades or same clinical stages. In patients with grade 3 ovarian cancer, patients with STIP1>169 (n = 102) had a significantly (P = 0.022) worse 5-year survival rate of 44.5% than 63.6% in those with STIP1≤169 (n = 11). In patients with grade 1–2 ovarian cancer, the cases with STIP1>169 (n = 62) had a worse 5-year survival rate of 73.8% than 85.3% in those with STIP1≤169 (n = 48), although the difference did not reach a statistical significance yet (P = 0.197). Furthermore, in the patients with advanced stages (III and IV) of serous carcinoma and undetermined grade 2–3 (n = 8), all of them had STIP1>169 and 7 of them (87.5%) succumbed to the disease during the follow up of this study.

**Figure 3:**
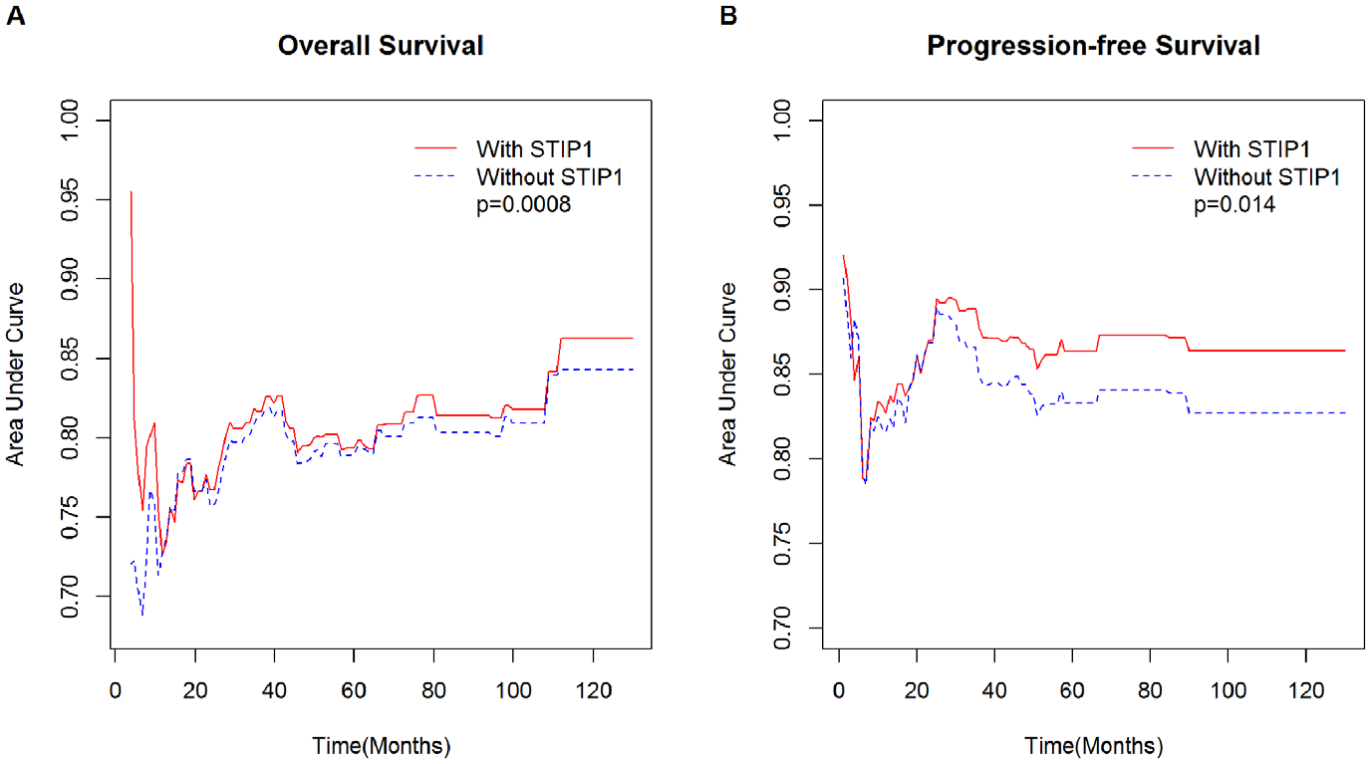
The addition of STIP1 histoscores improves the prediction of patients’ outcomes. The time-dependent AUC (area under the ROC curve) of the model including STIP1 is reported as a red solid line, whereas the AUC of the model without STIP1 is shown as a blue dotted line. Other parameters included stage, type, serum CA125, and residual disease. (A) Overall survival, (B) Progression-free survival.

We also performed an analysis of deviance and constructed the time-dependent ROC curves (either with or without STIP1) to examine whether the addition of STIP1 histoscores could improve the prognostic stratification of patients with invasive ovarian cancers. In these analyses, other potential predictors included in the model were stage, type, serum CA125, and the presence of residual disease. The model of adding the STIP1 histoscore to other prognostic factors significantly outperformed the model without STIP1, showing both higher AUCs and lower deviances in both OS (**Figure 3A, ***P* = 0.0008) and PFS (**Figure 3B, ***P* = 0.014). Collectively, these results indicated that tumor STIP1 histoscoring had a prognostic value in addition to the grading and staging.

### STIP1 Stimulated the Proliferation and Migration of Ovarian Cancer Cells

**Figure 4:**
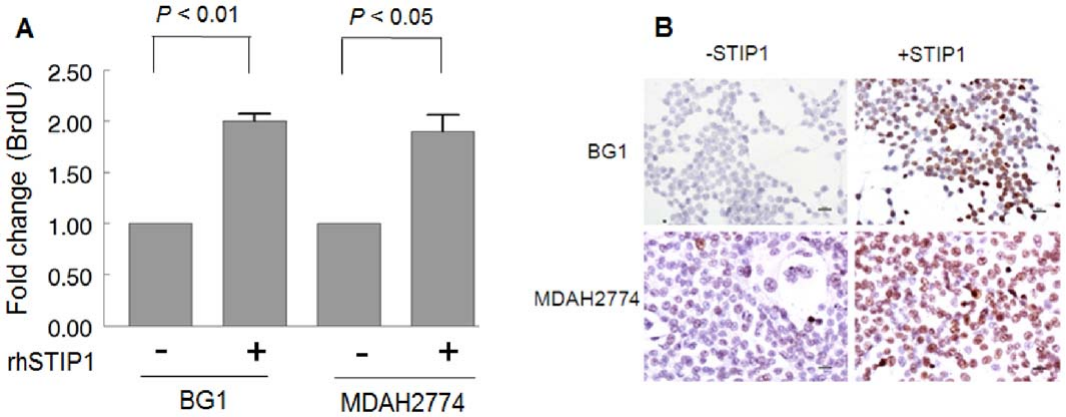
The treatment of ovarian cancer cells with recombinant human STIP1 (rhSTIP1) stimulates cell proliferation. Two ovarian cancer cell lines (BG1 and MDAH2774) were treated with 400 nM of rhSTIP1 and analyzed by BrdU incorporation (A) and Ki67 immunostaining (B). Ki67-positive cells are shown in brown color; scale bars represent 20 µ.

To investigate the biological functions of STIP1, we treated BG1 and MDAH2774 ovarian cancer cells with rhSTIP1 (400 nM). The treatment with rhSTIP1 induced a 1.9-fold stimulation in BrdU incorporation in both ovarian cancer cells (**Figure 4A** **)**. Moreover, the treatment with rhSTIP1 resulted in an increased Ki-67 immunostaining, suggesting that STIP1 induces human ovarian cancer cell proliferation (**Figure 4B**).

**Figure 5:**
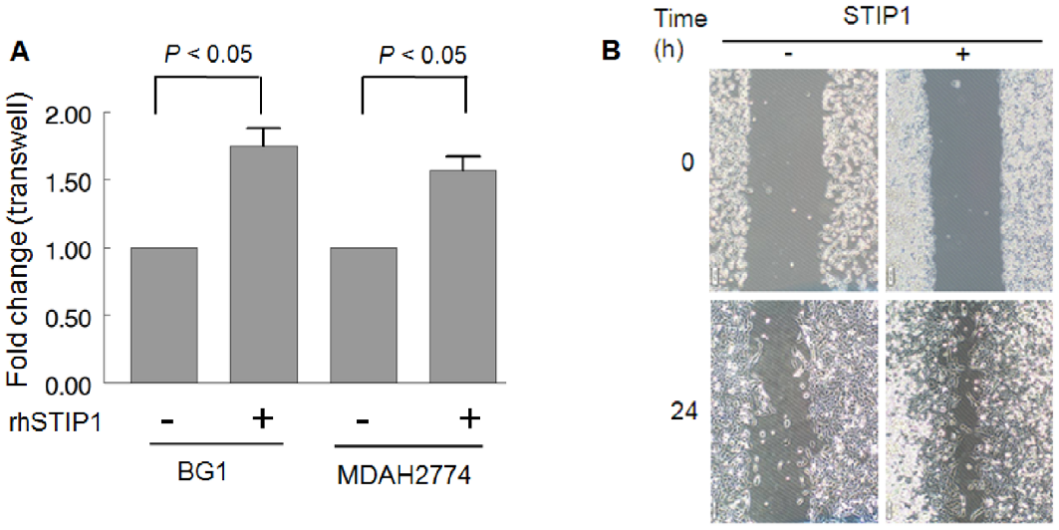
The treatment of ovarian cancer cells with recombinant human STIP1 (rhSTIP1) stimulates cell migration. The treatment with rhSTIP1 promoted cell migration according to the results of both Transwell migration (A) and wound closure (B) assays. Quantitative analysis of the percentage of migrating MDAH2774 cells using the WimScratch software showed that exposure to rhSTIP1 induced a 1.8-fold increase in migration (B). Results are representative of three independent experiments.

The treatment with rhSTIP1 significantly stimulated ovarian cancer cell migration (2.4-fold increase in BG1 cells and 1.6-fold increase in MDAH2774 cells, respectively) (**Figure 5A**). In agreement with the data obtained in the transwell migration assay, the results of the wound closure assay (**Figure 5B** **)** analyzed using the WimScratch software indicated a 1.8-fold increase in the migration rate of MDAH2774 cells treated with rhSTIP1 (data not shown).

**Figure 6:**
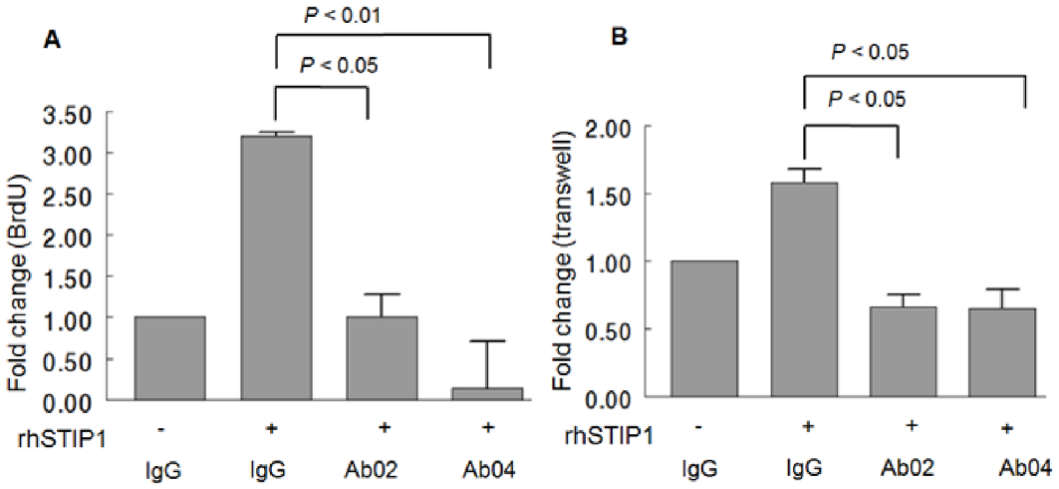
The effects of recombinant human STIP1 (rhSTIP1) on ovarian cancer cells are neutralized by the co-treatment with anti-STIP1 antibodies. When anti-STIP1 antibodies (800 nM) were added to cancer cells previously exposed to 400 nM of rhSTIP1, cell proliferation (A) and migration (B) were inhibited. Data are presented as means ± standard errors of the means of three independent experiments.

Importantly, the observed increases in cell proliferation and migration induced by rhSTIP1 were abrogated by the co-treatment with anti-STIP1 antibodies (**Figures 6A and 6B**). These results not only confirm that the observed effects were induced by STIP1, but also provide preliminary evidence to support the therapeutic potential of anti-STIP1 antibodies in ovarian cancer.

In ovarian cancer cells treated with rhSTIP1, siRNA knockdown of STIP1, or anti-STIP1 antibodies, we also used MTT assays to evaluate cell viability as a proxy for the cell number, with the understanding that cell number is shaped by the forces of both cell proliferation and apoptosis. In agreement with the results in **Figure 4** and **Figure 6A**, treatment with rhSTIP1 significantly (P<0.01) increased ovarian cancer cell number based on the MTT assay (**Figure S3A**), whereas knockdown of STIP1 significantly (P<0.005) suppressed ovarian cancer cells (**Figure S3B**). In contrast to the neutralizing effect of anti-STIP1 on the rhSTIP1-stimulated cell proliferation (**Figure 6A**), direct treatment of ovarian cancer cells with various clones of anti-STIP1 did not change the MTT readings (**Figure S3C**).

## Discussion

This study shows for the first time that STIP1 is a prognostic biomarker in human ovarian cancer. According to their individual molecular features, biomarkers have been recently grouped into the following categories: carcinogenesis biomarkers, released biomarkers, response biomarkers, and risk biomarkers [11]. Based on their application in disease characterization, biomarkers can also be classified as prognostic, predictive, and risk markers [19]. In our previous studies [9], [10], we have demonstrated that STIP1 is secreted by ovarian cancers in the bloodstream, suggesting that this molecule may serve as a released biomarker of ovarian cancer. Notably, our present findings indicate that a high STIP1 expression is related to poor OS (**Figure 2** **;** **Table 2**) and poor PFS rates (**Table 2**) and may thus represent a novel prognostic marker.

An important prognostic factor in ovarian cancer is the histological grade (**Figure 2B**); unfortunately, grading may be subjective even among experienced pathologists and some tumors do not fit neatly into a given grade [26]. For example, a significant inter-observer variation amongst pathologists in the grading of breast cancer has been reported [27]. To address this caveat, the classification of breast cancer into molecular subtypes with distinctive gene expression signatures has been proposed as a means for understanding the molecular basis of the histological grade [28]. In this study, we have shown that the STIP1 histoscores may be useful in supplementing the pathologist’s histopathological grading of ovarian cancers by providing objective, quantitative assessments. In particular, STIP1 histoscores may prove useful for predicting prognosis in patients with clear cell cancer (which is usually not graded) [3]. Our analyses further revealed that, even in patients of the same tumor grade, a higher STIP1 level was associated with a poorer clinical outcome.

Serum STIP1 levels were not shown to significantly differ among patients with 4 clinical stages of ovarian cancer [9], but in this study tumor STIP1 histoscores were significantly higher in stages III–IV (n = 147) than stages I–II (n = 183) (**Table 1**). This discrepancy may be merely caused by the small case number (n = 43) in our previous study [9]. The findings that high STIP1 histoscores were significantly associated with high clinical stages (III–IV) and high grade (3) (**Table 1**) also raised the possibility that high histoscores may be a function of high tumor grades and/or high clinical stages, which remains to be proved in future studies of larger case number. Nevertheless, tumor STIP1 histoscoring clearly exerts a prognostic value in addition to the commonly used, clinical parameters (**Figure 3**). These results collectively support the usefulness of STIP1 histoscore as a prognostic biomarker.

As a phosphoprotein, STIP1 undergoes a cdc2 kinase phosphorylation, which is accompanied by the cytoplasmic translocation of STIP1 [14]. The expression of STIP1 in various types of cancer [15], [29], [30], [31] suggests that this molecule has an anti-apoptotic role and/or promotes cancer cell survival. The knockdown of STIP1 has been shown to suppress the invasiveness of pancreatic cancer cells [31]. We have previously reported that STIP1 is secreted by ovarian cancer cells in the tumor microenvironment and the systemic circulation [9]. We have also described the molecular mechanisms by which secreted STIP1 stimulates the proliferation of ovarian cancer cells [10]. Results of the present study (**Figures 4** **and** **5**) not only confirm the role of STIP1 in promoting tumorigenesis but also suggest that this molecule may serve as a prognostic biomarker (**Figures 2** **and** **3** **,** **Table 2**).

The effective blockade of rhSTIP1-stimulated cell proliferation and migration of ovarian cells elicited by anti-STIP1 antibodies (**Figure 6**) indicates that secreted STIP1 from cancer tissues can be a target for the development of therapeutic antibodies in ovarian cancer. STIP1 represents an attractive candidate for cancer therapy. For example, a compound that prevents Hsp90 from interacting with STIP1 has been recently designed and shown to impair the Hsp90-dependent folding pathway, ultimately exerting cytotoxic effects on breast cancer cells [32]. Moreover, a novobiocin-derived Hsp90 C-terminal inhibitor, KU135, has been shown to inhibit proliferation and induce apoptosis in melanoma cells [33]. Finally, an *in vitro* study by Horibe et al. [34] has reported that an anti-TPR peptide that blocks the interaction of Hsp90 with the TPR2A domain of STIP1 is capable of inducing cell death in pancreatic, renal, lung, prostate, and gastric cancer cell lines.

Despite the incremental improvements in surgery and chemotherapy, the majority of patients with ovarian cancer die of the disease within five years of diagnosis [3]. For suitable patients, adding targeted therapy to chemotherapy may increase both the chance that the tumor would respond well to treatment as well as increase the duration that the tumor can be suppressed; together, this may lead to some prolonging of life. If corroborated by other studies, our data suggest that STIP1 may serve as a promising target for antibody-based ovarian cancer therapy.

## Supporting Information

Figure S1 **Specificity of the anti-STIP1 antibody in STIP1 recognition.** Twenty µg of protein lysate from each of ovarian cancer cell lines (serous SKOV3 cells; endometrioid TOV112D and MDAH2774 cells; clear cell cancer TOV21G and ES2 cells) were analyzed for STIP1 with the identical anti-STIP1 antibody (Abnova) that was used for immunohistochemistry throughout this study. Specificity of this antibody was confirmed by the detection of a single band at 65 kD (STIP1) throughout the proteins ranging from 35 to 180 kD.
(DOC)

Figure S2 **Patients with high cancer grade were associated with a reduced overall survival.** Kaplan-Meier curves for overall survival showed that women with grade 3 tumors (red line) had a significantly lower overall survival than those with grade 1–2 malignancies (blue line; log-rank test, *P*<0.0001). Since clear cell cancer and borderline ovarian tumor are not graded; subjects with clear cell cancer (n = 57) and borderline ovarian tumor (n = 50) were excluded from this study.
(DOC)

Figure S3 **MTT assays on ovarian cancer cells. (A)** Ovarian cancer MDAH2774 cells were treated with 400 nM of rhSTIP1 for 24 h before MTT assays were done. (B) STIP1 in ovarian cancer MDAH2774 and BG1 cells were knocked down with siRNA technology, and MTT assays were done. (C) Ovarian cancer MDAH2774 cells were directly treated with 800 nM of various clones of anti-STIP1 or control antibodies for 24 h before MTT assays were done. Data presented as mean ± S.E. were derived from 3 independent experiments.
(DOC)
