# Supplementary material for: Tumor Stress-Induced Phosphoprotein1 (STIP1) as a Prognostic Biomarker in Ovarian Cancer
Source: PLoS One. 2013 Feb 27;8(2):e57084. doi: 10.1371/journal.pone.0057084 (PMC3584135; doi:10.1371/journal.pone.0057084)
Supplement: Figure S1 — Specificity of the anti-STIP1 antibody in STIP1 recognition. Twenty µg of protein lysate from each of ovarian cancer cell lines (serous SKOV3 cells; endometrioid TOV112D and MDAH2774 cells; clear cell cancer TOV21G and ES2 cells) were analyzed for STIP1 with the identical anti-STIP1 antibody (Abnova) that was used for immunohistochemistry throughout this study. Specificity of this antibody was confirmed by the detection of a single band at 65 kD (STIP1) throughout the proteins ranging from 35 to 180 kD. (DOC) [file pone.0057084.s001.doc]

**Supporting Information**

**Tumor Stress-induced Phosphoprotein1 (STIP1) as a Prognostic Biomarker in Ovarian Cancer**

Angel Chao^1*^, Chyong-Huey Lai^1^, Chia-Lung Tsai^1^, Swei Hsueh^2^, Chuen Hsueh^2^, Chiao-Yun Lin^1^, Hung-Hsueh Chou^1^, Yu-Jr Lin^3^, Hsi-Wen Chen^4^, Ting-Chang Chang^1^, Tzu-Hao Wang^1,4,5*^

^1^Department of Obstetrics and Gynecology, Chang Gung Memorial Hospital and Chang Gung University, Taoyuan, Taiwan

^2^Department of Clinical Pathology, Chang Gung Memorial Hospital and Chang Gung University, Taoyuan, Taiwan

^3^Biostatistical Center for Clinical Research, Chang Gung Memorial Hospital, Taiwan

^4^Graduate Institute of Biomedical Sciences, Chang Gung University, Taiwan

^5^Genomic Medicine Research Core Laboratory, Chang Gung Memorial Hospital, Taoyuan, Taiwan


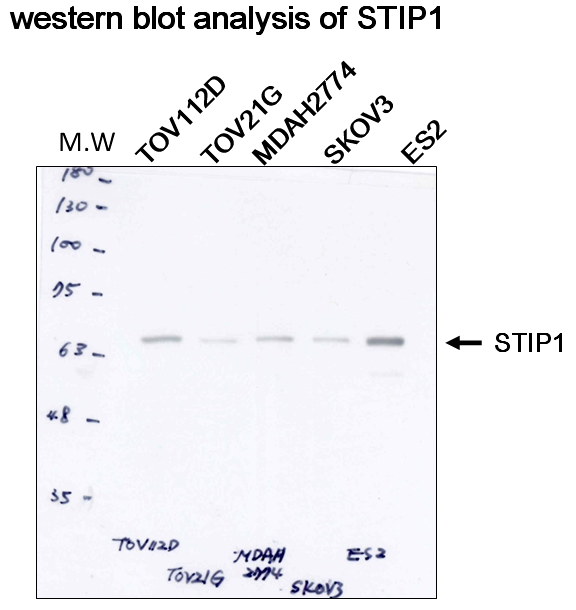


**Figure S1. Specificity of the anti-STIP1 antibody in STIP1 recognition.**

Twenty µg of protein lysate from each of ovarian cancer cell lines (serous SKOV3 cells; endometrioid TOV112D and MDAH2774 cells; clear cell cancer TOV21G and ES2 cells) were analyzed for STIP1 with the identical anti-STIP1 antibody (Abnova) that was used for immunohistochemistry throughout this study. Specificity of this antibody was confirmed by the detection of a single band at 65 kD (STIP1) throughout the proteins ranging from 35 to 180 kD.
