# Supplementary material for: Tumor Stress-Induced Phosphoprotein1 (STIP1) as a Prognostic Biomarker in Ovarian Cancer
Source: PLoS One. 2013 Feb 27;8(2):e57084. doi: 10.1371/journal.pone.0057084 (PMC3584135; doi:10.1371/journal.pone.0057084)
Supplement: Figure S2 — Patients with high cancer grade were associated with a reduced overall survival. Kaplan-Meier curves for overall survival showed that women with grade 3 tumors (red line) had a significantly lower overall survival than those with grade 1–2 malignancies (blue line; log-rank test, P<0.0001). Since clear cell cancer and borderline ovarian tumor are not graded; subjects with clear cell cancer (n = 57) and borderline ovarian tumor (n = 50) were excluded from this study. (DOC) [file pone.0057084.s002.doc]

**Supporting Information**

**Tumor Stress-induced Phosphoprotein1 (STIP1) as a Prognostic Biomarker in Ovarian Cancer**

Angel Chao^1*^, Chyong-Huey Lai^1^, Chia-Lung Tsai^1^, Swei Hsueh^2^, Chuen Hsueh^2^, Chiao-Yun Lin^1^, Hung-Hsueh Chou^1^, Yu-Jr Lin^3^, Hsi-Wen Chen^4^, Ting-Chang Chang^1^, Tzu-Hao Wang^1,4,5*^

^1^Department of Obstetrics and Gynecology, Chang Gung Memorial Hospital and Chang Gung University, Taoyuan, Taiwan

^2^Department of Clinical Pathology, Chang Gung Memorial Hospital and Chang Gung University, Taoyuan, Taiwan

^3^Biostatistical Center for Clinical Research, Chang Gung Memorial Hospital, Taiwan

^4^Graduate Institute of Biomedical Sciences, Chang Gung University, Taiwan

^5^Genomic Medicine Research Core Laboratory, Chang Gung Memorial Hospital, Taoyuan, Taiwan


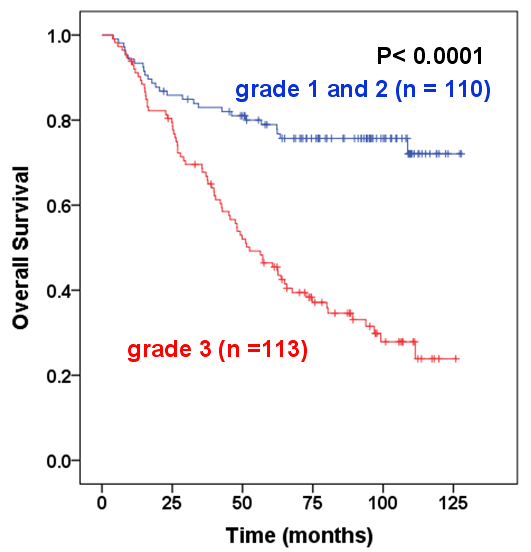


**Figure S2. Patients with high cancer grade were associated with a reduced overall survival.** Kaplan-Meier curves for overall survival showed that women with grade 3 tumors (red line) had a significantly lower overall survival than those with grade 1-2 malignancies (blue line; log-rank test, *P* < 0.0001). Since clear cell cancer and borderline ovarian tumor are not graded; subjects with clear cell cancer (n=57) and borderline ovarian tumor (n=50) were excluded from this study.
