# Supplementary material for: Tumor Stress-Induced Phosphoprotein1 (STIP1) as a Prognostic Biomarker in Ovarian Cancer
Source: PLoS One. 2013 Feb 27;8(2):e57084. doi: 10.1371/journal.pone.0057084 (PMC3584135; doi:10.1371/journal.pone.0057084)
Supplement: Figure S3 — MTT assays on ovarian cancer cells. (A) Ovarian cancer MDAH2774 cells were treated with 400 nM of rhSTIP1 for 24 h before MTT assays were done. (B) STIP1 in ovarian cancer MDAH2774 and BG1 cells were knocked down with siRNA technology, and MTT assays were done. (C) Ovarian cancer MDAH2774 cells were directly treated with 800 nM of various clones of anti-STIP1 or control antibodies for 24 h before MTT assays were done. Data presented as mean ± S.E. were derived from 3 independent experiments. (DOC) [file pone.0057084.s003.doc]

**Supporting Information**

**Tumor Stress-induced Phosphoprotein1 (STIP1) as a Prognostic Biomarker in Ovarian Cancer**

Angel Chao^1*^, Chyong-Huey Lai^1^, Chia-Lung Tsai^1^, Swei Hsueh^2^, Chuen Hsueh^2^, Chiao-Yun Lin^1^, Hung-Hsueh Chou^1^, Yu-Jr Lin^3^, Hsi-Wen Chen^4^, Ting-Chang Chang^1^, Tzu-Hao Wang^1,4,5*^

^1^Department of Obstetrics and Gynecology, Chang Gung Memorial Hospital and Chang Gung University, Taoyuan, Taiwan

^2^Department of Clinical Pathology, Chang Gung Memorial Hospital and Chang Gung University, Taoyuan, Taiwan

^3^Biostatistical Center for Clinical Research, Chang Gung Memorial Hospital, Taiwan

^4^Graduate Institute of Biomedical Sciences, Chang Gung University, Taiwan

^5^Genomic Medicine Research Core Laboratory, Chang Gung Memorial Hospital, Taoyuan, Taiwan

**A**

**
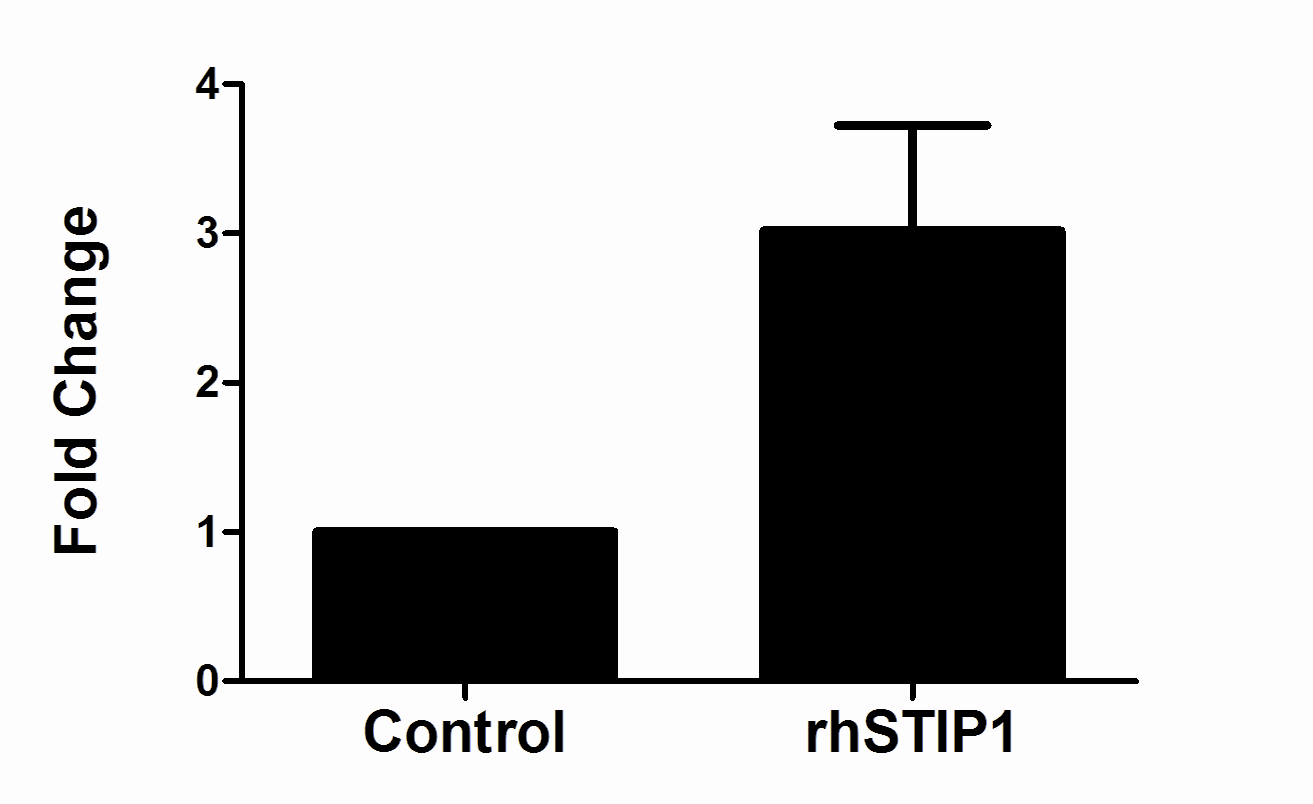
**

**B**

**
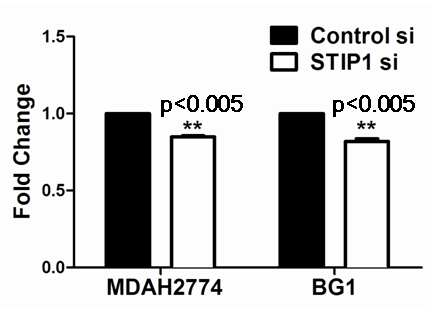
**

**C**

**
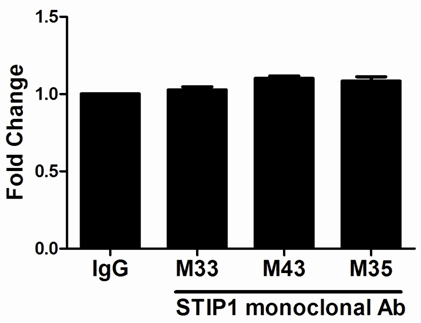
**

**Figure S3. MTT assays on ovarian cancer cells. (A)** Ovarian cancer MDAH2774 cells were treated with 400 nM of rhSTIP1 for 24 h before MTT assays were done. (B) STIP1 in ovarian cancer MDAH2774 and BG1 cells were knocked down with siRNA technology, and MTT assays were done. (C) Ovarian cancer MDAH2774 cells were directly treated with 800 nM of various clones of anti-STIP1 or control antibodies for 24 h before MTT assays were done. Data presented as mean ± S.E. were derived from 3 independent experiments.
